# A new PacBio genome sequence of an Australian *Pyrenophora tritici*-*repentis* race 1 isolate

**DOI:** 10.1186/s13104-019-4681-6

**Published:** 2019-10-04

**Authors:** Paula Moolhuijzen, Pao Theen See, Caroline S. Moffat

**Affiliations:** Centre for Crop Disease and Management, School of Molecular Life Sciences, Curtin University, Perth, Australia

**Keywords:** *Pyrenophora tritici*-*repentis*, Genome assembly, PacBio sequel, Comparative analysis, ToxA, Tan spot, Yellow spot

## Abstract

**Objectives:**

The necrotrophic fungal pathogen *Pyrenophora tritici*-*repentis* (Ptr) is the causal agent of tan spot a major disease of wheat. We have generated a new genome resource for an Australian Ptr race 1 isolate V1 to support comparative ‘omics analyses. In particular, the V1 PacBio Biosciences long-read sequence assembly was generated to confirm the stability of large-scale genome rearrangements of the Australian race 1 isolate M4 when compared to the North American race 1 isolate Pt-1C-BFP.

**Results:**

Over 1.3 million reads were sequenced by PacBio Sequel small-molecule real-time sequencing (SRMT) cell to yield 11.4 Gb for the genome assembly of V1 (285X coverage), with median and maximum read lengths of 8959 bp and 72,292 bp respectively. The V1 genome was assembled into 33 contiguous sequences with a of total length 40.4 Mb and GC content of 50.44%. A total of 14,050 protein coding genes were predicted and annotated for V1. Of these 11,519 genes were orthologous to both Pt-1C-BFP and M4. Whole genome alignment of the Australian long-read assemblies (V1 to M4) confirmed previously identified large-scale genome rearrangements between M4 and Pt-1C-BFP and presented small scale variations, which included a sequence break within a race-specific region for *ToxA,* a well-known necrotrophic effector gene.

## Introduction

The necrotrophic fungal pathogen *Pyrenophora tritici*-*repentis* (Ptr) is the causal agent of tan (or yellow) spot disease of wheat (*Triticum aestivum*), which has a significant economic impact on the grain industry worldwide [1]. Ptr, a necrotrophic fungal pathogen is an ascomycete within the order Pleosporales, which also contains other important crop pathogens [2, 3]. The production of necrotrophic host-specific effectors contributes to the pathogenicity of this fungus, and three Ptr effectors have been described to date. ToxA and ToxB, are both well-characterised small effector proteins that produce necrosis and chlorosis symptoms respectively [4, 5], while ToxC, which also causes chlorosis, remains to be identified and may be the product of a secondary metabolite gene cluster [6].

The *ToxA* gene, believed to have been horizontally transferred to Ptr from *Parastagonospora nodorum* [7], can occupy different loci positions in different isolates and races of Ptr [8]. It has been proposed that this type of translocation could be the result of gene proximity to a chromosomal break point [9]. Recently, we confirmed large scale chromosomal rearrangements/fusions between two race 1 isolates sourced from North America (Pt-1C-BFP) [10] and Australia (M4) [11]. We therefore undertook PacBio Biosciences (PacBio) Sequel system for long-read sequencing of a second Australian Ptr isolate (V1), collected from a different geographic state, for whole genome comparison necessary to confirm these larger rearrangements and examine any further genomic variations. Furthermore, a high quality PacBio genome assembly enables the characterization of predicted secondary metabolite gene clusters and effectors often contained in highly complex genomic regions.

## Main text

### Methods

#### Isolate collection and sequencing

The pathogenic isolate V1 was isolated from tan spot infected leaves collected from Horsham, Victoria, Australia in 2015. V1 was cultured in vitro from a single spore [12].

V1 genomic DNA was extracted from 3-day old mycelia grown in vitro in Fries 3 liquid medium, using a BioSprint 15 DNA Plant Kit (Qiagen, Hilden, Germany) and automated workstation according to the manufacturer’s instruction [11]. DNA was further treated with 50 μg/ml of RNase enzyme (Qiagen, Hilden, Germany) for 1 h followed by phenol/chloroform extraction, followed by precipitation with sodium acetate and ethanol, and finally resuspension in TE buffer [11].

The V1 genome was sequenced using PacBio Biosciences (PacBio) Sequel small-molecule real-time sequencing (SMRT) Cell Technology at 283X to yield 11.4 Gb (1,319,569 long reads) by Novogene Co., Ltd, Hong Kong. The V1 genome was also sequenced via Illumina HiSeq 150 bp PE by Novogene Co., Ltd (Hong Kong) to yield 3.2 Gb at 80X coverage. Illumina read Phred quality score distribution was greater than 30, and reads containing adaptors were removed.

#### Plant materials and pathogenicity assays

To assess pathogenicity and race classification differential wheat genotypes, which differ in their effector sensitivities were used for inoculation [5, 13]. The wheat lines used were Glenlea and BG261 (both ToxA-sensitive), 6B662 (ToxB-sensitive) and 6B365 (ToxC-sensitive). Two week-old wheat (*Triticum aestivum* L.) seedlings were spore-inoculated by spraying the whole plants evenly with approximately 2000 conidia/ml and grown under controlled growth conditions [14]. The second leaves were harvested 7-days post-inoculation, visually inspected for symptoms and photographed. Infection experiments were repeated twice with four replicate plants per wheat line to demonstrate the pathogenicity and race classification.

#### Genome assembly of V1 and comparative analysis

PacBio sequence data was error-corrected and assembled using linux-amd64 Canu 1.8 software [15] guided by a genome size of 40 Mb. Illumina PE reads were quality trimmed for random hexamer primers on the 5′ read end using Trimmomatic v0.22 [16]. The high quality trimmed Illumina reads were aligned to the Canu genome assembly using BWA 0.7.14-r1138 [17] and filtered for concordant PE read alignments using samtools 0.1.19-96b5f2294a [18]. The Canu genome assembly was additionally corrected with the high quality Illumina alignments using Pilon 1.2 [19] to generate a final polished V1 sequence assembly with SNP and INDEL corrections.

V1 was then aligned to Pt-1C-BFP [10] and M4 [11] scaffolded chromosomes using NUCmer v3.1 (-maxmatch -coords). The sequence alignment for Pt-1C-BFP (NCBI Accession DS231618.1), M4 (chr6) and V1 (C11 reverse complemented and C12 reverse complemented) was conducted using Progressive Mauve 2015-02-25 [20], and EasyFig Version 2.2.3 [21], BLASTN, filter 500 bp length.

#### Gene prediction and functional annotation

M4 Illumina RNA-seq data [11] was aligned to V1 using TopHat v2.0.12 [22] (-N 2 -i 10 -I 5000 -p 16 –no-discord- ant –no-mixed –report-secondary-alignments –micro- exon-search –library-type fr-firststrand) for supporting ab initio gene predictions by CodingQuarry v1.2 [23] in pathogen mode (PM). Ab initio gene predictions were also made with GeneMark-ES v4.33 [24].

Pt-1C-BFP and M4 reference proteins [10] [11] were aligned to V1 using Exonerate v2.2.0 [25] (–showvulgar no –showalignment no –minintron 10 –maxintron 3000) in mode protein2genome. The ab initio gene predictions and exonerate alignments were then combined using EvidenceModeller v1.1.1 [26] with a minimum intron length of 10 bp and weightings of CodingQuarry:1, GeneMark.hmm:1, protein exonerate:2.

Gene annotations were assigned by BLASTX v2.2.26 searches across NCBI RefSeq and NR (taxon = Ascomycota) (October 2018) databases and RPS-BLAST v2.2.26 of Pfam, Smart and CDD domain databases (October 2018). Final gene annotations were summarised by AutoFACT v3.4 [27].

Pt-1C-BFP, M4 and V1 proteins were clustered to identify orthologous genes using OrthoFinder version 1.1.4 [28]. Predicted gene proteins for isolates Pt-1C-BFP, M4 and V1 were also assessed using BUSCO version3 [29] against Fungi OrthoDB version 9.

### Results

**Fig. 1:**
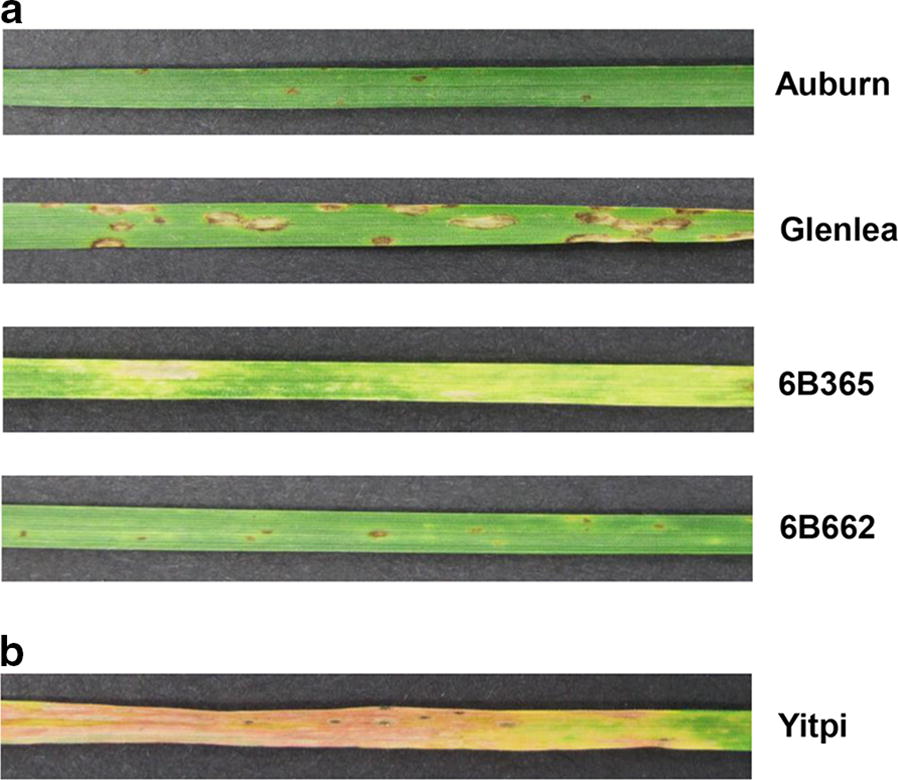
Pathogenicity assays of V1. **a** The presence of symptoms on the differential wheat lines Glenlea (ToxA sensitive) and 6B365 (ToxC sensitive), and the absence of tan spot symptoms on Auburn (insensitive) and 6B662 (ToxB sensitive) confirms a race 1 (ToxA and ToxC) classification for V1. **b** Susceptible disease reaction displayed on the Australian commercial wheat variety, Yitpi

Plant infection assays confirmed V1 as a race 1 isolate (producing ToxA and ToxC), by the presence of necrosis and chlorosis symptoms on the differential wheat lines Glenlea and 6B365 respectively, and absence of tan spot symptoms on Auburn and 6B662 (Fig. 1a). Furthermore, the induced chlorosis symptoms on the tan spot susceptible Australian commercial wheat cultivar Yitpi, also confirmed V1 pathogenicity (Fig. 1b).

The genomic sequence of V1 was assembled into 33 contiguous sequences with a of total length of 40,408,077 bp and a N50 of 3,421,861 bp. The mean and longest contig sizes were 1,224,487 bp and 9,664,470 bp respectively. V1 contig length statistics showed an improvement in comparison to M4 and Pt-1C-BFP (Table 1). A total of 14,050 protein coding genes were annotated for V1, which included the major effector *ToxA* (PtrV1_13859) positioned on contig12: 1,348,464–1,349,050. A total of 10,398 genes were orthologous to Pt-1C-BFP and M4. The V1 annotated genome has been deposited with National Center for Biotechnology Information (NCBI) GenBank under the accession SAXQ00000000.

**Table 1:** *Pyrenophora tritici*-*repentis* race 1 isolate genome information and assembly statistics

	V1	M4^a^	Pt-1C-BFP^a^
Isolate information			
Sequencing Platform	PacBio Sequel	PacBio RSII	Sanger, Illumina
Genome accession	SAXQ00000000	NQIK00000000	AAXI00000000
Collection site	Victoria, Australia	Western Australia, Australia	South Dakota, USA
Collection year	2015	2009	1994
Contig assembly statistics			
Total length (Mb)	40.4	40.9	38.0
Number	33	51	48
N50 (Mb)	3.4	2.9	1.9
Mean (Kb)	1224	802	778
Max (Mb)	9.6	5.6	6.7
GC %	50.4	50.7	50.8
Gene predictions			
Number	14,050	13,797	12,171
%^b^BUSCO gene	96.55	98.62	98.62

^a^Previously published genome assemblies

^b^Complete and fragmented gene

**Fig. 2:**
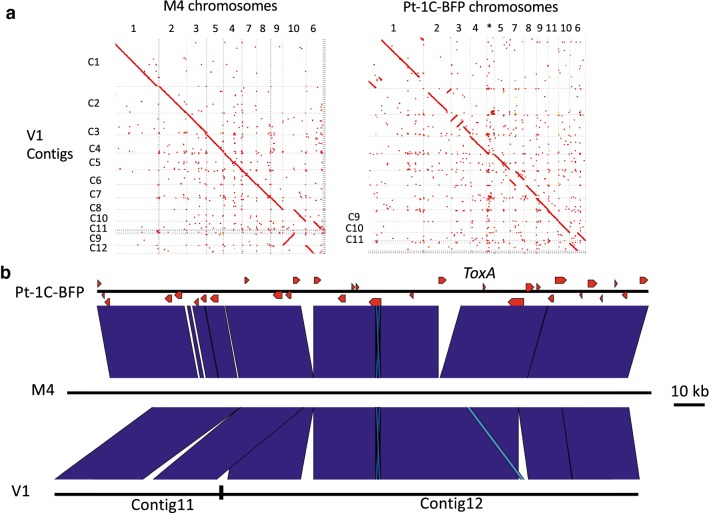
**a** Dot plots showing the whole genome nucleotide alignments for V1 contigs (vertical axis) to M4 and Pt-1C-BFP scaffolded genomes (horizontal axes). *Pt-1C-BFP contigs that are not assigned to a Pt-1C-BFP chromosome. **b** Sequence alignment showing Pt-1C-BFP Chr6:1.32–1.50 Mb, M4 Chr6:1.57–1.75 Mb and V1 contigs11 and 12 (both reverse complemented). The V1 contig break point is indicated 76 kb upstream of the *ToxA* locus (red arrow labelled as *ToxA*)

Whole genome alignment of V1 to M4 (PacBio RSII) and Pt-1C-BFP (Sanger) confirmed the same large-scale rearrangements in V1 that were found in M4 for chr3, chr7 and the distal regions of chr1 and 2 when compared to Pt-1C-BFP (Fig. 2a). However, V1 had sequence breaks at points not observed for M4 chr6 and observed for chr10 (which were confirmed previously by M4 optical mapping) [11]. The sequence alignment of M4 (chr6:1.57–1.75 Mb), Pt-1C-BFP (chr6 1.32–1.50) and V1 (contigs 11 and 12) highlight the sequence break point in V1 76,077 bp upstream of the *ToxA* effector gene (Fig. 2b), that was not found in M4.

### Discussion

The PacBio sequence for V1 had longer assembly statistics when compared to the recent M4 PacBio RSII (six SMRT cells) assembly, possibly due to the higher depth of coverage obtained from the PacBio Sequel system for overlap assembly.

The sequence comparison of V1 to both M4 and Pt-1C-BFP confirmed the chromosomal rearrangements for M4 chr1, chr2, chr3 and chr7 for the Australia isolates, which appears stable despite isolates being collected from different geographic states. Smaller scale differences between V1 and M4 were however detected in chr4, chr6, chr7 and chr8. Also, V1 had a sequence break upstream of *ToxA* with a significant sequence variation between Pt-1C-BFP and M4 in both length and complexity that was not resolved by assembly. These larger and smaller scale rearrangements can impact gene clusters, especially when they are proximal to complex sub-telomeric regions and breakpoints. This resource will therefore be useful for future ‘omics experiments and comparative Ptr genomic analyses.

## Limitations

Although all methods have been made as consistent as possible for comparative analyses, this analysis has used databases, software and PacBio sequencing versions currently available, which may be updated in the future. The comparison of two Australian long-read assemblies is only an indication of potential genome stability in Australia.

## Data Availability

All data generated or analysed during this study are included in this article. The assembled and annotated genome for isolate V1 was submitted to NCBI GenBank repository under accession SAXQ00000000.

## Abbreviations

Ptr: *Pyrenophora tritici*-*repentis*
PacBio: PacBio Biosciences
SRMT: small-molecule real-time sequencing
NCBI: National Centre for Biotechnology Information
